# Angiotensin-Converting Enzyme Genotype and Peripheral Arterial Disease in Diabetic Patients

**DOI:** 10.1155/2012/698695

**Published:** 2011-11-14

**Authors:** Chin-Hsiao Tseng, Farn-Hsuan Tseng, Choon-Khim Chong, Ching-Ping Tseng, Ju-Chien Cheng

**Affiliations:** ^1^Department of Internal Medicine, National Taiwan University College of Medicine, Taipei 100, Taiwan; ^2^Division of Endocrinology and Metabolism, Department of Internal Medicine, National Taiwan University Hospital, Taipei 100, Taiwan; ^3^Department of Biological Sciences, Dana and David Dornsife College of Letters, Arts and Sciences, University of Southern California, Los Angeles, CA 90089, USA; ^4^Chong's Physical Medicine and Rehabilitation Center, Taipei 116, Taiwan; ^5^Department of Medical Biotechnology and Laboratory Science, Chang Gung University, Taoyuan 333, Taiwan; ^6^Department of Medical Laboratory Science and Biotechnology, China Medical University, Taichung 404, Taiwan

## Abstract

We investigated the effect of traditional risk factors (hypertension, dyslipidemia and smoking) on the association between angiotensin-converting enzyme (ACE) gene insertion/deletion (I/D) polymorphism and peripheral arterial disease (PAD) in 945 (454 men and 491 women) Taiwanese type 2 diabetic patients with a mean age of 63.5 (SD: 11.4) years. Among them, 81 (31 men and 50 women) had PAD (ankle-brachial index <0.9). The adjusted odds ratios (95% confidence intervals) were 2.48 (1.18–5.21), 1.69 (1.00–2.85) and 1.64 (1.12–2.39), respectively, for recessive (DD versus II + ID), dominant (DD + ID versus II) and additive (II = 0, ID = 1 and DD = 2) models. While analyzing the interaction between DD and the individual risk factor of hypertension, smoking and dyslipidemia, patients with the risk factor and with DD had the highest risk compared to referent patients without the risk factor and with II/ID. The respective adjusted odds ratios were 5.41 (2.05–14.31), 7.38 (1.87–29.06) and 4.64 (1.70–12.64). We did not find a significant interaction between DD and any of the risk factors under multiplicative or additive scale. In conclusion, traditional risk factors (hypertension, smoking and dyslipidemia) play an important role in the association between ACE genotypes and PAD. Patients with DD genotype and traditional risk factors are at the highest risk.

## 1. Introduction

Angiotensin-converting enzyme (ACE) converts angiotensin I to angiotensin II and degrades bradykinin [1]. Its activity is genetically determined by an insertion/deletion (I/D) polymorphism [2]. Individuals with DD genotype, a marker for diabetic nephropathy [1], hypertension [1], renal artery stenosis [3], cardiomyopathies [1], and coronary and carotid atherosclerosis [1], have a twofold increase in ACE concentration [2]. However, its potential as a risk factor for peripheral arterial disease (PAD) is rarely studied.

Smoking plays an important role in mediating the association between ACE polymorphism and the intima media thickness of the carotid arteries [4], coronary atherosclerosis [5], and cardiovascular mortality [6]. A meta-analysis confirmed the association between DD and carotid intima media thickness, which was more prominent in high-risk populations [7]. These observations supported the importance of traditional risk factors together with ACE gene on cardiovascular disease. However, studies simultaneously evaluating the effects of traditional risk factors including hypertension, dyslipidemia, and smoking on the association between the ACE genotypes and PAD are still lacking. The present study assessed the interaction between these risk factors and DD genotype on PAD risk in Taiwanese patients with type 2 diabetes mellitus.

## 2. Materials and Methods

### 2.1. Subjects

The study was approved by an ethics committee, and all subjects participated with their informed consent. A total of 945 (454 men and 491 women) diabetic patients were recruited consecutively from the outpatient clinics of a medical center in Taiwan. The mean age was 63.5 (SD: 11.4) years. To avoid recruitment of patients with type 1 diabetes mellitus, the patients must be treated with oral antidiabetic drugs or insulin, and they must not have received insulin treatment within one year of diabetes diagnosis, nor showed diabetic ketoacidosis at disease onset. We chose type 2 diabetic patients for the study because the prevalence of PAD is low in the general population. Even in the high risk diabetic patients, the prevalence of PAD is only approximately 10% in our population [8].

### 2.2. Diagnosis of PAD

Diagnosis of PAD was based on an ankle-brachial index (ABI) <0.9 on either side of the lower extremities as described in our previous studies [8–10]. Doppler ultrasound (Medacord PVL, MedaSonic Inc., Mountain View, Calif, USA) was used to measure the systolic blood pressure on bilateral brachial, posterior tibial and dorsal pedal arteries on a supine position after a 20-minute rest. The occluding cuffs (55 cm × 12.5 cm) were applied just above the malleoli for measurement of ankle pressures. The Doppler probe used was 8 MHz in frequency. Right and left ABI were calculated automatically by the device by dividing the higher pressure on the dorsal pedal or posterior tibial arteries on right and left sides, respectively, by the higher brachial pressure on either side.

### 2.3. Determination of Risk Factors and Covariates

Smoking, hypertension, and dyslipidemia were investigated for their interaction with ACE genotypes. Uric acid [8] and lipoprotein (a) [9] have been identified as significant predictors for PAD in our diabetic patients. Therefore, the confounders included age, sex, body mass index, duration of diabetes, hemoglobin A_1c_, uric acid, and lipoprotein (a). 

Ever smokers (*n* = 333) were defined as those who smoked one or more cigarettes per day. Patients who did not smoke were defined as never smokers (*n* = 611). Blood pressure was measured on the right arm after 20 min rest on a sitting position with a mercury sphygmomanometer. The first perception of successive sounds was taken as systolic blood pressure and the complete disappearance of sound (Korotkoff phase V) as diastolic blood pressure. Patients were defined as having hypertension if undergoing antihypertensive treatments, having systolic blood pressure ≥140 mmHg, or showing diastolic blood pressure ≥90 mmHg. 

Total cholesterol, triglycerides, high- and low-density lipoprotein cholesterol, uric acid, lipoprotein (a), and hemoglobin A_1c_ were determined [8–10]. Dyslipidemia was defined as triglycerides ≥150 mg/dL and/or high-density lipoprotein cholesterol <35 mg/dL for men or <39 mg/dL for women, and/or total cholesterol ≥200 mg/dL, and/or low-density lipoprotein cholesterol ≥100 mg/dL, or those undergoing treatment for lipid disorder [11]. 

Duration of diabetes was defined as the time period in years between the time being recruited into the study and the time diabetes was diagnosed. Body weight and body height were measured with light clothes and bare feet. Body mass index was calculated as body weight in kilograms divided by the square of body height in meters.

### 2.4. Genomic DNA Preparation and ACE Genotyping

Genomic DNA was extracted from peripheral blood, and the genotyping of the ACE gene was performed as described previously [12]. Briefly, the extracted DNA was subject to polymerase chain reaction (PCR) using sense oligo 5′-CTGGAGACCACTCCCATCCTTTCT-3′ and antisense oligo 5′-GATGTCGCCATCACATTCGTCAGAT-3′ as primers in a solution containing 1.5 mmol/L MgCl_2_, 50 mmol/L KCl, 10 mmol/L Tris-HCl, 0.1% (w/v) gelatin, 1% Triton X-100, 0.3 mmol/L each of dNTP (ProTech), and 2 units Pro Taq DNA polymerase (ProTech), with pH 9.0. Approximately 500 ng to 1 *μ*g of genomic DNA was used per reaction. The PCR cycling was performed with initial denaturation at 94°C for 3 min followed by 35 cycles of 94°C for 1 min, 58°C for 1 min, 72°C for 2 min, and then by a final extension period at 72°C for 7 min. The PCR products were either 490 base pairs (bps) (insertion allele) or 190 bps (deletion allele). The D allele is preferentially amplified in heterozygotes giving rise to mistyping of ID as DD in approximately 5% of cases [13]. To avoid such mistyping, samples of DD genotype were subject to a second independent PCR with primers that recognize an insertion-specific sequence: 5′-TGGGACCACAGCGCCCGCCACTAC-3′ as sense and 5′-TCGCCAGCCCTCCCATGCCCATAA-3′ as antisense primers. The PCR reaction yields a 335 bp DNA product in the presence of the I allele [13].

### 2.5. Statistical Analyses

The PC version of Statistical Package for Social Sciences (SPSS 10.0, Chicago, Ill, USA) was used. Data were expressed as means (SD) and case number (percentages). Triglycerides and lipoprotein (a) were logarithmically transformed due to skewness. *P* < 0.05 was considered statistically significant. Deviations from the Hardy-Weinberg equilibrium were assessed with chi-square test. Student's *t*-test or one-way ANOVA and chi-square test were used to compare the differences of the continuous and categorical variables including the differences among the ACE genotypes, respectively. 

The following gene transmission models were considered: (1) a recessive effect (DD versus ID + II), (2) a dominant effect (DD + ID versus II), and (3) an additive effect (assigning 0, 1, and 2 for II, ID, and DD, respectively) of the D allele. The association was evaluated by chi-square test followed by logistic regression after adjustment for age, sex, body mass index, duration of diabetes, hemoglobin A_1c_, uric acid, ln[lipoprotein (a)], hypertension, smoking, and dyslipidemia. The Akaike information criterion (AIC = −2 × model log-likelihood + 2 × number of model parameters) was computed, and the model with lowest AIC was considered the best fit. Models differing in AIC by <2 units were considered indistinguishable [14]. 

To assess the interaction between risk factor and DD, the two-by-four table [15] and the synergy indices of multiplicativity (SIM) and additivity (SIA) [16] were applied. The patients were divided into 4 subgroups: (A) without risk factor (hypertension, smoking, or dyslipidemia) and without DD as referent group; (B) without risk factor but with DD, (C) with risk factor but without DD, and (D) with risk factor and with DD. Multiple logistic regression was used to estimate the adjusted odds ratios for PAD among the 4 subgroups (treated as a categorical variable in the model) using patients without the risk factor and without DD as referent. These models were created after adjustment for age, sex, body mass index, duration of diabetes, hemoglobin A_1c_, uric acid, ln[lipoprotein (a)], and the two risk factors other than the one used for classifying the patients (e.g., in the models classifying the patients according to hypertension and ACE genotypes, smoking and dyslipidemia were additionally adjusted). Additional logistic regression was performed to calculate the synergy indices by entering the covariates, the risk factors, the ACE genotype, and the product term of risk factors (one at a time) and ACE genotype as independent variables. SIM was the odds ratio of the product term. SIA was computed according to Zou [16].

## 3. Results

The allele frequencies for I and D were 69.9% and 30.1%, respectively. The frequencies of II, ID, and DD were 49.0%, 41.9%, and 9.1%, respectively, with the distribution in the Hardy-Weinberg equilibrium. The case number for those without any risk factors of smoking, hypertension, or dyslipidemia was 101; with only one risk factor was 58, 119, and 131, respectively; with two risk factors of smoking and hypertension, smoking and dyslipidemia, or hypertension and dyslipidemia was 55, 95, and 260, respectively; with all three risk factors was 125 (smoking not available in one case). 

Table 1 compares the characteristics of patients with and without PAD. Age, duration of diabetes, body mass index, hypertension, systolic blood pressure, dyslipidemia, ln(triglycerides), ln[lipoprotein (a)], and uric acid were significantly different, but DD genotype and D allele frequencies were not.

Table 2 compares the characteristics by genotypes. Even though the point estimate for PAD prevalence in those with DD was higher, the prevalence did not differ significantly from that in the II and ID group.

Table 3 shows the gene transmission models. In chi-square test, none were significant. However, the adjusted odds ratios for recessive and additive models were significant, while that for the dominant model was borderline significant with *P* value of 0.050. AIC was lowest for the additive model in the adjusted regressions. However, the adjusted additive and recessive models were indistinguishable because they differed in AIC by <2 units. 

Table 4 shows the gene-environment interactions. In the multiple logistic regression models with patient categorization based on two-by-four table, the subgroups with the risk factor and DD showed the highest risk; the subgroups without the risk factor but with DD were not significantly different from the referents. All SIMs and SIAs did not differ from unity and suggested a lack of interaction on either the multiplicative or the additive scale.

## 4. Discussion

This is probably the first study evaluating the interaction between individual risk factor of hypertension, smoking, or dyslipidemia and the ACE DD genotype on the risk of PAD by classifying the patients using the two-by-four table and by using the synergy indices for evaluation of gene-environment interaction. We demonstrated that the predisposition of DD to PAD was greatest in the presence of risk factors including hypertension, smoking, or dyslipidemia, and observed that there was no significant interaction on the scale of either additivity or multiplicativity. 

In contrast to a stronger association between DD and disease in low-risk subjects [17] or a lack of such a subgroup association [18], this study strongly suggested that PAD risk increased significantly in the presence of both traditional risk factors and DD. Two decades ago, Khoury et al. asserted that failure to consider the effect of environmental factors can result in an estimation of the relative risk close to unity implying no genetic effect even if there is a strong gene-environment effect [19]. Accordingly we did not observe a significant association between DD and PAD in crude analyses (Tables 1 and 2). However, when the combination effects of traditional risk factors and DD genotype were considered, PAD risk increased significantly (Table 4). Our present study can be viewed as a case study supporting the assertion made by Khoury et al. [19]. 

Studies with smaller sample sizes tended to report larger odds ratios [18]. One reason is that the cases were more correctly diagnosed and the controls better selected in smaller studies. Other explanations include publication bias and occurrence by chance. The larger odds ratios in our study might be due to selection bias resulting from a hospital-based design, the smaller sample sizes in the subgroups with DD and PAD, the more accurate diagnosis of PAD with the Doppler ultrasound or the more accurate genotyping by confirmatory PCR. Because the genotype distributions were in the Hardy-Weinberg equilibrium, the possibility of selection bias was reduced. Due to the small sample size in the subgroups with DD and PAD, we were unable to evaluate with sufficient statistical power the simultaneous interactions among multiple risk factors and DD by dividing the subjects into more subgroups. We also recognized that diabetes mellitus is a risk factor and all subjects were having at least this one risk factor, even in the referent groups. Therefore our findings should be reconfirmed and the joint effects of multiple risk factors should be evaluated by future population-based studies with larger sample sizes collected ideally in a prospective fashion.

Based on the following reasons we believed that the association is not spurious. First, the strength of association was strong and consistent. Second, a Bonferroni's correction for 12 tests still yielded significant *P* values for patients with DD and the risk factor (Table 4). Third, the results were not influenced if the models were adjusted for the following additional confounders one at a time: smoking in pack-years, use of antihypertensive agents, number of antihypertensive agents used (as a proxy for the severity of hypertension), and use of lipid-lowering agents (data not shown). 

Although we have additionally analyzed the use of antihypertensive and lipid-lowering drugs in secondary analyses, we still could not completely exclude the possible residual confounding effects of medications used by the patients over time which might not have been captured at the time of the study. Furthermore, the effects of many risk factors might be cumulative over time and the lack of their analyses as time varying variables was a possible limitation. A residual confounding by the severity of diabetes might also be a concern because, though not statistically significant, patients with DD had a higher hemoglobin A_1c_ (Table 2). The number of patients with PAD (*n* = 81) in comparison to those without PAD (*n* = 864) reflected the prevalence of PAD in this cohort of diabetic patients. The PAD prevalence of 8.6% in this study is in line with earlier studies conducted in Taiwan [8–10]. However, the relatively small number of PAD cases may indicate that the study was underpowered. Another limitation of the study was that we did not measure ACE levels for the patients and could not evaluate the association among PAD risk, DD genotype, and ACE level.

In summary, Taiwanese patients with type 2 diabetes mellitus who simultaneously carry the DD genotype and possess the individual risk factors of hypertension, smoking or dyslipidemia are at especially high risk of PAD.

## Figures and Tables

**Table 1:** Comparisons of characteristics of study subjects with and without peripheral arterial disease.

Characteristics	Peripheral arterial disease		*P*
	Yes	No	
*n*	81	864	
Age, years	72.9 (7.3)	62.6 (11.3)	<0.001
Sex			
Men	31 (38.3)	423 (49.0)	0.066
Women	50 (61.7)	441 (51.0)	
Duration of diabetes, years	16.3 (9.3)	10.8 (7.7)	<0.001
Body mass index, kg/m^2^	23.6 (3.3)	24.9 (3.4)	0.001
Smoking			
Never smokers	51 (63.0)	560 (64.9)	>0.1
Current or former smokers	30 (37.0)	303 (35.1)	
Hypertension			
No	17 (21.0)	369 (42.7)	<0.001
Yes	64 (79.0)	495 (57.3)	
Systolic blood pressure, mmHg	146.0 (18.2)	134.0 (17.3)	<0.001
Diastolic blood pressure, mmHg	82.9 (12.0)	83.4 (9.6)	>0.1
Hemoglobin A_1c_, %	7.9 (1.6)	7.9 (1.9)	>0.1
Dyslipidemia			
No	18 (22.2)	316 (36.6)	0.010
Yes	63 (77.8)	548 (63.4)	
Total cholesterol, mg/dL	209.7 (42.2)	205.1 (43.3)	>0.1
Ln(triglycerides), mg/dL	5.1 (0.6)	5.0 (0.6)	0.03
High-density lipoprotein cholesterol, mg/dL	46.8 (12.7)	48.1 (13.6)	>0.1
Low-density lipoprotein cholesterol, mg/dL	115.5 (34.9)	114.2 (33.8)	>0.1
Ln[lipoprotein (a)], mg/dL	5.0 (1.2)	4.7 (1.1)	0.011
Uric acid, mg/dL	5.9 (2.2)	5.2 (2.0)	0.006
Angiotensin-converting enzyme genotype			
II	35 (43.2)	430 (49.8)	>0.1
ID	34 (42.0)	358 (41.4)	
DD	12 (14.8)	76 (8.8)	
Allele frequency			
I	104 (64.2)	1,218 (70.5)	>0.1
D	58 (35.8)	510 (29.5)	

Data are expressed as mean (SD) or *n* (percentage).

**Table 2:** Comparisons of characteristics by insertion/deletion (I/D) polymorphism of angiotensin-converting enzyme genotypes.

Characteristics	Angiotensin-converting enzyme genotype		
	II	ID	DD
*n* (%)	465	392	88
Age, years	64.1 (11.7)	62.8 (11.2)	63.4 (11.0)
Sex			
Men	219 (47.1)	192 (49.0)	43 (48.9)
Women	246 (52.9)	200 (51.0)	45 (51.1)
Duration of diabetes, years	11.8 (8.2)	10.7 (7.9)	10.8 (7.6)
Body mass index, kg/m^2^	24.8 (3.6)	24.8 (3.3)	25.0 (3.6)
Smoking			
Never smokers	309 (66.6)	244 (62.2)	58 (65.9)
Current or former smokers	155 (33.4)	148 (37.8)	30 (34.1)
Hypertension			
No	191 (41.1)	158 (40.3)	37 (42.0)
Yes	274 (58.9)	234 (59.7)	51 (58.0)
Systolic blood pressure, mmHg	135.8 (18.1)	133.9 (16.6)	135.5 (19.5)
Diastolic blood pressure, mmHg	83.3 (9.9)	83.2 (9.5)	84.3 (10.7)
Hemoglobin A_1c_, %	7.8 (1.8)	7.9 (1.9)	8.2 (1.9)
Dyslipidemia			
No	167 (35.9)	132 (33.7)	35 (39.8)
Yes	298 (64.1)	260 (66.3)	53 (60.2)
Total cholesterol, mg/dL	204.3 (43.1)	208.3 (43.8)	199.5 (40.3)
Ln(triglycerides), mg/dL	5.0 (0.6)	5.0 (0.6)	4.9 (0.6)
High-density lipoprotein cholesterol, mg/dL	47.4 (13.5)	48.9 (13.6)	46.7 (12.9)
Low-density lipoprotein cholesterol, mg/dL	114.1 (34.7)	115.4 (34.2)	110.5 (27.6)
Ln[lipoprotein (a)], mg/dL	4.7 (1.2)	4.7 (1.1)	4.6 (1.2)
Uric acid, mg/dL	5.2 (2.1)	5.3 (1.9)	5.3 (2.1)
Peripheral arterial disease			
No	430 (92.5)	358 (91.3)	76 (86.4)
Yes	35 (7.5)	34 (8.7)	12 (13.6)

Data are expressed as mean (SD) or *n* (percentage).

None of the above comparisons among the genotypes was statistically significant (i.e., all *P* > 0.05).

**Table 3:** Gene transmission models for insertion/deletion (I/D) polymorphism of angiotensin-converting enzyme gene on peripheral arterial disease.

	chi-square test				Logistic regression*		
Gene transmission	Total *N*	Peripheral arterial disease, *n* (%)		*P* value	Adjusted odds ratio (95% confidence interval)	*P* value	Akaike information criterion
		No	Yes				
Recessive model							
II/ID	857	788 (91.9)	69 (8.1)		1.00		
DD	88	76 (86.4)	12 (13.6)	0.075	2.48 (1.18–5.21)	0.017	425.880
Dominant model							
II	465	430 (92.5)	35 (7.5)		1.00		
ID/DD	480	434 (90.4)	46 (9.6)	0.259	1.69 (1.00–2.85)	0.050	427.157
Additive model							
II = 0,	465	430 (92.5)	35 (7.5)		1.00		
ID = 1,	392	358 (91.3)	34 (8.7)		1.64 (1.12–2.39) per risk		
DD = 2	88	76 (86.4)	12 (13.6)	0.171	allele	0.010	424.612

*Adjusted variables include age, sex, body mass index, duration of diabetes, hemoglobin A_1c_, uric acid, ln[lipoprotein (a)], hypertension, smoking, and dyslipidemia.

**Table 4:** Gene-environment models evaluating the interaction between insertion/deletion (I/D) polymorphism of angiotensin-converting enzyme (ACE) genotypes and traditional risk factors including hypertension, smoking, and dyslipidemia in peripheral arterial disease.

Risk factor	ACE genotype	Total *N*	Peripheral arterial disease, *n* (%)		Adjusted odds ratio (95% confidence interval)	*P*-value	Synergy index of multiplicativity	Synergy index of additivity
			No	Yes				
Hypertension								
No	II/ID	349	334	15	1.00		1.23 (0.19–8.01)	2.01 (0.32–12.8)
	DD	37	35	2	2.10 (0.40–11.17)	0.383		
Yes	II/ID	508	454	54	2.09 (1.07–4.07)	0.030		
	DD	51	41	10	5.41 (2.05–14.31)	0.001		

Smoking								
Never smokers	II/ID	553	510	43	1.00		1.21 (0.25–5.87)	2.16 (0.43–10.80)
	DD	58	50	8	2.33 (0.94–5.81)	0.069		
Ever smokers	II/ID	303	277	26	2.63 (1.16–5.96)	0.021		
	DD	30	26	4	7.38 (1.87–29.06)	0.004		

Dyslipidemia								
No	II/ID	299	284	15	1.00		1.21 (0.23–6.37)	1.88 (0.29–12.04)
	DD	35	32	3	2.16 (0.53–8.90)	0.285		
Yes	II/ID	558	504	54	1.77 (0.92–3.43)	0.088		
	DD	53	44	9	4.64 (1.70–12.64)	0.003		
